# An on-chip electrical transport spectroscopy approach for *in situ* monitoring electrochemical interfaces

**DOI:** 10.1038/ncomms8867

**Published:** 2015-08-06

**Authors:** Mengning Ding, Qiyuan He, Gongming Wang, Hung-Chieh Cheng, Yu Huang, Xiangfeng Duan

**Affiliations:** 1 Department of Materials Science and Engineering, University of California, Los Angeles, California 90095, USA; 2 California Nanosystems Institute, University of California, Los Angeles, California 90095, USA; 3 Department of Chemistry and Biochemistry, University of California, Los Angeles, California 90095, USA

## Abstract

*In situ* monitoring electrochemical interfaces is crucial for fundamental understanding and continued optimization of electrocatalysts. Conventional spectroscopic techniques are generally difficult to implement for *in situ* electrochemical studies. Here we report an on-chip electrical transport spectroscopy approach for directly probing the electrochemical surfaces of metallic nanocatalysts in action. With a four-electrode device configuration, we demonstrate that the electrical properties of ultrafine platinum nanowires are highly sensitive and selective to the electrochemical surface states, enabling a nanoelectronic signalling pathway that reveals electrochemical interface information during in-device cyclic voltammetry. Our results not only show a high degree of consistency with generally accepted conclusions in platinum electrochemistry but also offer important insights on various practically important electrochemical reactions. This study defines a nanoelectronic strategy for *in situ* electrochemical surface studies with high surface sensitivity and surface specificity.

## Abstract (web summary)

*In situ* probing electrochemical interfaces is important for the development of improved electrocatalysts. Here, the authors present an on-chip electrical transport spectroscopy approach, which enables *in situ* monitoring the dynamic electrochemical interface characteristics.

## Graphical abstract

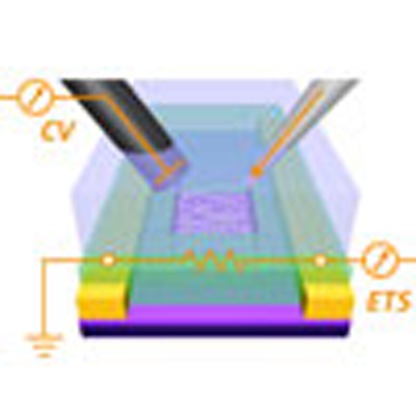

Electrochemistry of catalytic nanomaterials has played an essential role in diverse energy-related technologies, including fuel cells^1^,^2^,^3^, batteries^4^ and many other electro/photocatalytic processes^5^,^6^,^7^,^8^. A fundamental understanding of the electrocatalytic surfaces/interfaces is crucial for developing future generation of nanocatalysts. To this end, *in situ* electrochemical surface study is most informative^9^, yet extremely challenging because these interfaces are generally buried between solid support and liquid electrolyte and are thus difficult to access by the conventional spectroscopic techniques. Considerable efforts have been attempted for *in situ* probing electrochemical interfaces using thin-layer cells^10^ or synchrotron radiation^11^, which typically requires unusual device design or highly specialized resources^12^,^13^,^14^. Therefore, it is of considerable significance to develop novel signalling pathways and analytic tools, alternative to the traditional spectroscopic techniques, that allow for *in situ* probing the electrochemical interfaces in action.

With reducing physical dimension and increasing surface-to-bulk ratio in ultrafine metal nanostructures, their electrical properties could be greatly influenced by the surface conditions. For example, it is well-known that upon molecular adsorption, the resistance of a metallic thin film increases due to the increased diffusive scattering of conduction electrons (that is, the electron-free path is terminated by the collision at the surface, as opposed to specular scattering, where electron scattered at the surface is specularly reflected)^15^,^16^. The surface scattering effect of adsorbed molecules on ultrafine metallic nanostructure has been explored for nanoelectronic chemical detectors^17^,^18^,^19^ and biosensors^20^. In these nanodevices, the change of electrical conductivity of a metallic nanostructure served as a signal pathway to the surface adsorption of selected chemical/biological species. Here we demonstrate, for the first time, the use of such nanoelectronic signalling pathway to probe the dynamic electrochemical interfaces for nanoscale electrocatalysts. With a properly designed nanodevice based on the network of ultrafine platinum nanowires (PtNWs), in-device cyclic voltammetry (CV) of PtNWs is performed with concurrent *in situ* measurement of their electrical conductivity. This method allows us to use the nanoelectronic device as an electronic probe, alternative to the spectroscopic probes, for *in situ* monitoring the dynamic electrochemical interface characteristics between the metallic nanostructures and electrolyte under variable electrochemical conditions. A differentiation method is further developed for a more convenient and efficient visualization and analysis of the electrical transport characteristics. A systematic analysis of the electrical signals of PtNWs shows a high degree of consistency with the generally accepted conclusions in the field of Pt electrochemistry. More significantly, it reveals important new insights on various Pt-catalysed electrochemical reactions.

## Results

### Underlying principles

**Figure 1:**
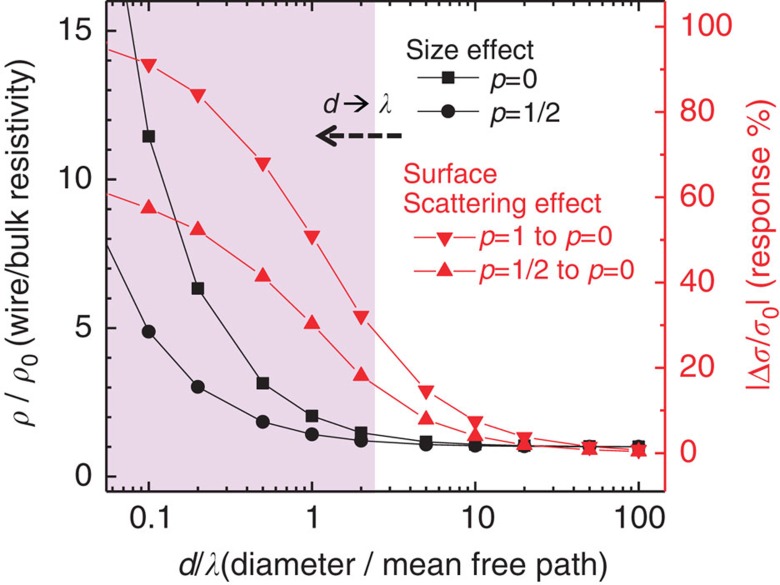
Theoretical size and surface scattering effect of metallic nanowires. Black squares (for *p*=0) and circles (for *p*=1/2) depict the size dependence of the resistivity for one-dimensional cylindrical metallic nanowires. The resistivity (over bulk metal resistivity) increases markedly when the nanowire diameter approaches to the electron mean free path. Triangles depict the size dependence of surface scattering induced conductivity response. The relative conductivity change upon adsorption (which induces surface scattering and causes changes in *p* value) increases markedly when the nanowire diameter approaches to the corresponding electron mean free path. *ρ*(*σ*), resistivity (conductivity) of the metal thin wire; *ρ*_*0*_, resistivity of the bulk metal; *d*, diameter of the metal thin wire; *λ*, mean free path of the conduction electrons; *p*, portion of electrons specularly reflected on the metal surface.

In general, the surface scattering induced resistance change in one-dimensional cylindrical wires can be calculated using the same equations that are used to describe the size dependence of metals^21^:


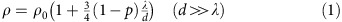


and


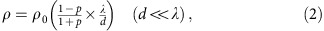


where *ρ* is the resistivity of metallic wire, *ρ*_*0*_ is the resistivity of bulk metal, *λ* is the corresponding electron mean free path, *d* is the wire diameter and *p* is the portion of conduction electrons specularly reflected on the metal surface. Molecules adsorbed on metal nanostructure can function as diffusive scattering centre, resulting in a reduced *p* value and increased resistivity (*ρ*)^16^,^17^. It is important to note that for a specific electron mean free path in a given metal, the response signal (Δ*ρ/ρ*_*0*_) is inversely proportional to the dimension of metallic nanostructures. Therefore, with reducing diameter in ultrafine metallic nanowires (especially when *d* approaches *λ*), the diffusive scattering induced by surface adsorption can produce a significant change in resistivity (or conductivity *σ*). Figure 1 shows that the surface scattering induced signal (Δ*σ/σ*_*0*_) of metallic nanowires derived using equations (1) and (2). It is evident that a substantial signal can be clearly seen when the nanowire diameter becomes comparable to or smaller than the electron mean free path.

### Device fabrication

**Figure 2:**
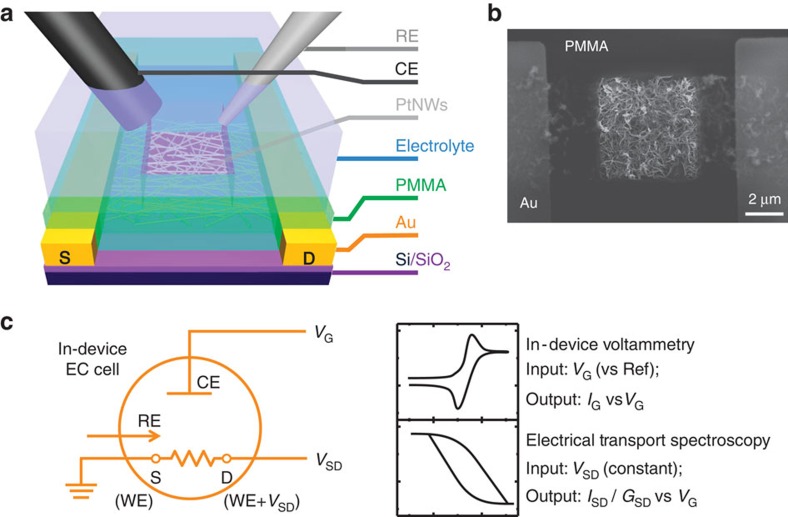
Working principle of *in situ* electrical transport spectroscopy (ETS) measurement. (**a**) Schematic illustration of the platinum nanowire (PtNW) device with a microscopic electrochemical (EC) cell on it. (**b**) Scanning electron microscopy image of a device cell showing PMMA covered gold electrodes, and the exposed PtNWs network in the opened PMMA window. Scale bar, 2 μm. (**c**) Schematic diagram of concurrent in-device CV and ETS for *in situ* monitoring of the electrochemical interfaces. CE, counter electrode; RE, reference electrode; WE, working electrode; S, source; D, drain.

Figure 2a summarizes the overall device configuration, while detailed description of device fabrication and electrochemical set-up can be found in Supplementary Methods and Supplementary Fig. 1. Briefly, ultrafine PtNWs with ∼2-nm diameters were synthesized^22^, assembled into a thin film using a co-solvent evaporation method^23^, and then selectively deposited onto the Si/SiO_2_ wafer with pre-patterned gold electrodes. An electrochemically inert layer (poly(methyl methacrylate, PMMA) was employed to isolate the electrodes and defining the electrochemical window (through electron-beam (e-beam) lithography). The morphology of the PtNWs network was examined by scanning electron microscopy (Fig. 2b) and other microscopic characterizations (see Supplementary Fig. 2).

### Working principle of ETS measurement

The general working principle that allowed in-device monitoring of the electrochemical interfaces is outlined in Fig. 2c. Our device configuration and measurement design resembles that of the electrolyte-gated semiconductor transistors. Instead of using electrolyte gating only to induce an electrical double layer on the semiconducting channel, three-electrode system was adopted and the gate voltage was extended to a range so that the desired electrochemical reactions could occur on PtNWs. Unlike semiconductor nanostructures that are highly sensitive to the electrochemical (gating) potential, the metallic nanostructures only respond to surface bound molecules through surface scattering (see Supplementary Figs 3, 4, 5 and 6 and Supplementary Discussion for detailed discussion on the origins of the signal in this measurement system). This characteristic minimizes the background signal from the electrolyte media or electrical potential variation in the CV process, and offers a robust signalling pathway that is only sensitive to surface conditions of the conducting channel (that is, the signal exhibit an exceptional surface specificity that is not available in semiconductor nanostructures).

In specific, a standard source–measure unit (SMU) was used to sweep the gate voltage (*V*_G_) between reference electrode and PtNWs, and to collect the Faradic current (gate current, *I*_G_) through counter electrode, functioning as a pseudo-potentiostat^18^ for in-device CV. A second SMU was used to simultaneously measure the electrical transport properties of PtNWs during the CV process. PtNWs were placed between two protected gold electrodes (source and drain), and a small constant bias voltage (*V*_SD_) was applied during the measurement. The source-drain current (*I*_SD_) or conductance (*G*_SD_) of PtNWs was recorded during the in-device CV, and their varying value in response to the sweeping *V*_G_ is an indicative parameter of the changing surface scattering effect at different electrochemical potentials, which can thus serve as an effective signal revealing the surface adsorption states and electrochemical interface characteristics during any specific electrochemical reactions. We have designated these electrical transport-based analyses of electrochemical interfaces as electrical transport spectroscopy (ETS).

### In-device CV and *in situ* ETS results

**Figure 3:**
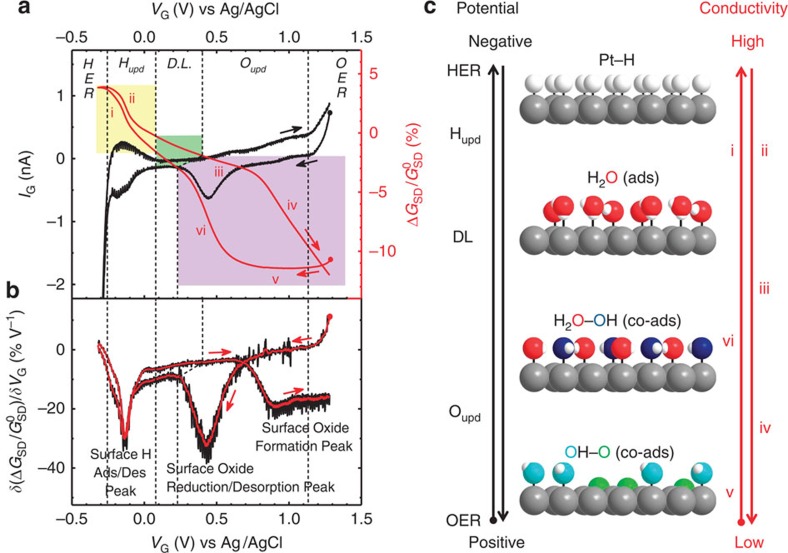
*In situ* electrical transport spectroscopy (ETS) of the PtNWs. (**a**) *I*_G_*–V*_G_ (black curve) and normalized *G*_SD_*–V*_G_ (red curve) characteristics of a typical PtNWs device. *I*_G_*–V*_G_ resembles the typical CV characteristic of a polycrystalline Pt surface, containing redox regions of hydrogen evolution reaction (HER), H adsorption/desorption region (H_upd_), double layer (DL) region, surface oxide formation/reduction region (O_upd_) and oxygen evolution reaction (OER). *G*_SD_–*V*_G_ (ETS) curve can also be divided into three regions marked with yellow, green and purple boxes correspondingly. (**b**) Differentiated ETS (DETS) curve shows spectral peak characteristics. Red curve is a 15-point-averaged result for visual guide of the differentiated data. (**c**) Schematic illustrations of different Pt surface conditions with the sweeping electrochemical potentials (left black axis) and the corresponding conductivity changes (right red axis, labels shown along the axis are corresponding to the labels shown on *G*_SD_ curve in **a**). Pt atoms are grey, H atoms are white, O atoms are red in H_2_O_ads_, blue in OH_ads_ and green in O_ads_ for visual guide to the different scattering effect. Arrows in all figures indicate the potential sweeping direction, with corresponding dots showing the starting point of measurement.

A typical in-device CV and *in situ* electrical transport signal of a PtNW device is shown in Fig. 3a. The *I*_G_*–V*_G_ result (black curve in Fig. 3a) highly resembles the typical CV characteristic of polycrystalline Pt surface^24^ and bulk PtNWs membrane^22^, containing five redox regions: hydrogen evolution reaction (HER); H adsorption/desorption region (H_upd_); double layer (DL) region ; surface oxide formation/reduction region (O_upd_); and oxygen evolution reaction (OER). This result demonstrates the validity of the in-device CV measurement (see Supplementary Fig. 7 for detailed discussion). Between the onset potentials of HER and OER, the obtained CV current (*I*_G_) is exclusively associated with specific surface adsorption/desorption processes, which can serve as a robust reference point for identifying the surface states of PtNWs at a given potential (*V*_G_). The red curve in Fig. 3a is derived from source-drain current (*I*_SD_) of PtNWs under different gate voltage (*V*_G_) that is measured concurrently with in-device CV (*I*_G_*–V*_G_). Here the *I*_SD_ value is normalized to obtain the relative conductance change (Δ*G*_SD_*/G*_SD_^0^, see Methods). To fully interpret the *G*_SD_*–V*_G_ curve (ETS signal), electrochemical processes on the Pt surface at each potential region and the corresponding surface scattering effect needs to be considered together.

On the basis of the nature of surface chemical process, the *G*_SD_*–V*_G_ curve of PtNWs depicted in Fig. 3a can be divided into three sections (marked with three colours), in correspondence with the three potential regions identified in the CV curve (*I*_G_*–V*_G_). First, the *G*_SD_ shows a relatively small change in the DL region (green box in Fig. 3a), where the surface of PtNWs is predominantly occupied by a layer of absorbed water molecules (in a DL model, this first layer of adsorbed water molecules defines the inner Helmholtz plan, see Supplementary Fig. 5).

Second, at the negative potential range, adsorption/desorption of a monolayer of H atoms on the PtNWs surface (H_ads_) results in an obvious increase (i)/decrease (ii) in *G*_SD_, respectively (yellow box in Fig. 3a), while the *G*_SD_ signal exhibits little change during the HER. The increase in *G*_SD_ during the H adsorption can be attributed to a weaker diffusive scattering of electrons by a Pt–H surface than a Pt–H_2_O surface. Similar phenomena have also been observed in the case of H_2_ gas adsorptions on Pt^17^,^25^. There is a relatively small hysteresis in *G*_SD_ for H adsorption (i) and desorption (ii) process, indicating a highly reversible electrochemical process.

Third, in the positive potential region corresponding to the adsorption/desorption of surface oxygenated species (indicated by CV current), a more pronounced *G*_SD_ signal (purple box in Fig. 3a) is observed along with much larger hysteresis (see Supplementary Fig. 8 for more discussion). In the positive scan, *G*_SD_ curve first goes through a gradual decrease (iii) followed by a much steeper decrease (iv). The gradual decrease in *G*_SD_ can be attributed to the adsorption of hydroxyl species (OH_ads_; see Supplementary Fig. 9), and the steep decrease at higher potential is associated with the surface oxide formation. The larger response for the oxide formation can be attributed to the larger scattering cross-section of the strongly bonded O atoms on the Pt surface and the partial surface composition transition from metallic Pt to Pt oxide, which significantly reduces the free electron density in PtNWs. The conductance of PtNWs remains relatively stable at low level when the voltage sweeping direction is reversed to negative (v), and only starts to increase (vi) after the onset of the reduction process (coinciding with a cathodic current peak in CV).

It is interesting to note that *G*_SD_ exhibits a continued decrease during the OER, in contrast to a relative flat *G*_SD_ behaviour during HER process. These different features suggest distinct Pt surface dynamics during HER and OER cycles. The relatively flat *G*_SD_ feature at HER indicates that a complete and stable monolayer H_ads_ is formed on the Pt surface at the onset of HER process; while the continued decrease in *G*_SD_ during OER process indicates that the surface oxide formation (or penetration of O species into the PtNWs) does not reach the maximum coverage at the onset of OER and the oxidation process continues in the OER region. Importantly, this observation invalidates the generally accepted hypothesis that the coverage of oxygenic species reached saturation at the onset of oxygen evolution^24^,^26^. These analyses demonstrate that the *in situ* ETS approach can be used to reveal new insight about electrochemical interfaces that is not readily available previously with other approaches.

It is noticed that the most informative ETS signals indicating the transition between different electrochemical interface conditions come from the variations (or trend) of *G*_SD_ rather than its absolute value. Therefore, a differentiation operation is applied to the *G*_SD_ data to better visualize and analyse such variation-based signals, which we designated as the differentiated ETS (DETS). A typical DETS curve (*δ*(Δ*G*_SD_/*G*_SD_^0^)/*δV*_G_) reveals a spectral line feature with several peaks (Fig. 3b). Indeed, each change of surface conditions on PtNWs, induced by electrochemical reactions during in-device CV, can be identified by a peak on the differentiation curve. The correlation between electronic signals of the device and the electrochemical surface states of the material is schematically illustrated in Fig. 3c. Importantly, the scattering-based mechanism provides a highly surface sensitive signalling pathway for probing the electrochemical interfaces in real time. From the signals acquired via the ETS approach (coupled with concurrent CV studies), we can derive a molecular level explanation of the CV characteristics that is consistent with the conclusions developed for Pt electrodes or electrocatalysts through decades of electrochemical and spectroscopic studies^24^. Moreover, the ETS signal observed in this study reveals important new insights on the electrochemical interfaces that are not readily available from traditional spectroscopic analysis. It can therefore function as a powerful *in situ* characterization approach for mechanistic investigation of the electrochemical surface properties in highly complex environment.

Except for a qualitative identification of the surface conditions of PtNWs that helps to interpret the ETS signal, the CV current (*I*_G_) can also be used for quantitative analysis when it is only associated with the surface process (such as in the case of H_upd_). For example, the surface coverage of H_ads_ (Θ_H_) on PtNWs is calculated from the integrated charge of CV current at H_upd_ region (from 0.1 to −0.25 V versus Ag/AgCl) in comparison with the CO stripping charge of PtNWs (see Supplementary Fig. 10). By such analysis, a quantitative correlation between the surface conditions and the *G*_SD_ signal can be established, which could serve as a calibration for the study of complicated electrochemical reactions using the *in situ* ETS approach.

### *In situ* ETS study of H_2_O_2_ reduction and oxidation reactions

To further demonstrate the significance of ETS, we have employed this technique to explore different electrochemical reactions catalysed by PtNWs. In these cases, cathodic/anodic currents from redox reactions dominate the voltammetric signals and thus little surface information could be directly derived from the conventional CV studies. To this end, the ETS approach can function as a highly surface-specific analysis technique to reveal important insight on electrochemical surface states during active electrochemical reactions.

**Figure 4:**
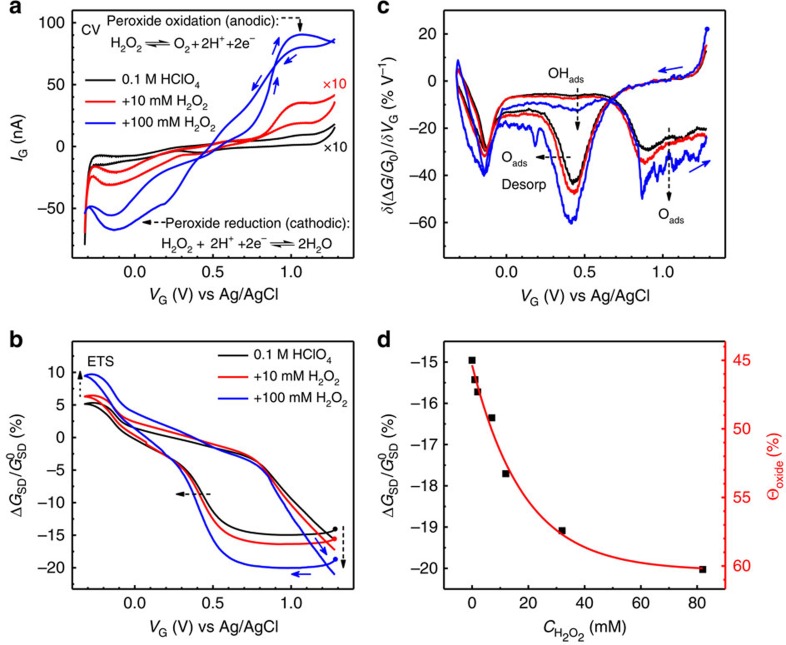
Hydrogen peroxide reduction and oxidation reaction (PROR) on PtNWs. (**a**,**b**) *I*_G_*–V*_G_ (**a**) and normalized *G*_SD_*–V*_G_ (**b**) characteristics of a typical PtNWs device during PROR at different H_2_O_2_ concentrations. *I*_G_*–V*_G_ (CV) characteristic was dominated by the cathodic/anodic current of PROR and therefore surface information of PtNWs was not directly accessible from the CV during the reaction. Dashed arrows in **b** indicate the shift of ETS characteristic during PROR. (**c**) Differentiated ETS (DETS) curves of PtNWs during PROR, dashed arrows indicate the shift of DETS characteristic during PROR. (**d**) *G*_SD_ value (black square) of the surface oxide for different H_2_O_2_ concentrations. Red curve is first order exponential fitting of the *G*_SD_ values, with estimated surface coverage of oxygenated species (Θ_Oxide_) at different H_2_O_2_ concentrations given at right axis. The Θ_Oxide_ for baseline (no H_2_O_2_) is obtained from integrated charges for surface OH adsorption (from 0.4 to 0.7 V versus Ag/AgCl) in comparison with CO stripping charge measurements. Solid arrows in all figures indicate the potential sweeping direction, with corresponding dots showing the starting point of measurement.

We have first studied the electrochemistry of hydrogen peroxide (H_2_O_2_) on PtNWs, because of following: (i) the similar chemical species involved in the H_2_O_2_ reduction and oxidation reactions (PROR) and (ii) the fundamental significance to understand the H_2_O_2_ electrochemistry on the model Pt catalysts, as it is identified as an important intermediate in oxygen reduction reactions (ORRs)^27^,^28^,^29^. We have conducted in-device CV (*I*_G_*–V*_G_) and ETS (*G*_SD_*–V*_G_) in perchloric acid with different concentrations of H_2_O_2_ (Fig. 4a,b, respectively). Result from PtNWs with no PROR is shown as a baseline (black curves in Fig. 4a,b). At high H_2_O_2_ concentrations, large PROR redox currents dominate the voltammogram and mask the intrinsic CV characteristic of PtNWs. For similar situations in the study of Pt electrocatalysis, information about the electrode surface, such as the surface coverage of O species at a given potential, could be estimated from the baseline cyclic voltammogram of electrode with no reactants, and the same conclusion was often applied in the discussion of polarization curves in PROR and ORR^1^,^27^. However, this assumption is not necessarily true since the electrochemical surface states could change under different reactant concentrations. Such information is nonetheless difficult to decouple from the cathodic/anodic current in traditional CV studies (as can be seen in Fig. 4a). Importantly, with the surface-specific nature of scattering-based mechanism, the *in situ* ETS reveals the information of Pt surface conditions during PROR processes at each different H_2_O_2_ concentrations, without being interfered by the faradic current through the gate channel. As shown in Fig. 4b, distinguishable *G*_SD_ signals were observed with different amount of H_2_O_2_, suggesting that the surface conditions of the Pt electrocatalysts can vary substantially during PROR. This observation provides evidence for an important question that has not been easily addressed in the related studies in PROR^27^ or ORR^1^.

The most significant change that can be identified in the *G*_SD_ results (Fig. 4b) is related to the adsorption of O species. We find this part of *G*_SD_ curves drops to a lower value with increasing H_2_O_2_ concentration. Given the origin of decreasing conductance due to surface oxidation, the greater decrease of the *G*_SD_ suggests a larger fraction of the oxidized Pt surface. This conclusion is not surprising considering H_2_O_2_ (or its cathodic product O_2_) is an oxidizing agent and higher H_2_O_2_ could lead to a higher O_ads_ coverage or a larger fraction of the surface oxide layer. The analysis of the DETS curve leads to the same conclusion (Fig. 4c): with increasing H_2_O_2_ concentrations, the O adsorption peak and O_ads_ desporption peak both show an increased intensity corresponding to the *G*_SD_ drop at the positive potential scan and subsequent increase at the negative scan. Moreover, it is evident that, with increasing H_2_O_2_ concentrations, the O_ads_ desorption peak is broadened to a more negative potential while the onset potential at the positive side remains the same (Fig. 4c). This peak broadening/shifting has also been observed in 0.1 M HClO_4_ with increasing upper potential limit (see Supplementary Fig. 8), as a result of the increased penetration of O atoms into the bulk Pt at higher upper potential limit, which requires a more negative potential to be reduced^24^. Similarly, the broadening/shifting of O_ads_ desorption peak at higher H_2_O_2_ concentration can be attributed to the more intensive O penetration into the PtNWs. It is important to note that in PROR, the CV signal is completely dominated by the H_2_O_2_ redox current and cannot reveal such Pt surface information. This is another example that the ETS signal not only gives the same electrode surface information as CV measurement in baseline conditions (such as in 0.1 M HClO_4_; Supplementary Fig. 8a,b) but also continues to reveal electrode surface information when the CV signal is completely dominated by the reactant redox currents (such as in PROR, Fig. 4a–c). Furthermore, an obvious increase in the intensity of the OH_ads_ peak was also observed, implying the increased coverage of adsorbed hydroxyl species during the cathodic oxidation of H_2_O_2_. Since the surface O_ads_ coverage is crucial to many catalytic processes including ORR^1^,^30^,^31^, this surface information revealed by ETS approach could provide valuable information to guide the rational design of optimized electrocatalysts.

For a more detailed understanding of electrocatalysis (for example, to probe reaction kinetics), quantitative information of the electrode surface is often needed. Up to date, the quantitative analysis of the electrode surface almost exclusively came from the faradic charge that can be possibly linked to the surface coverage of certain adsorbed chemical species. For traditional *in situ* spectroscopic studies, the spectroscopic signals may be calibrated with these redox charges when there is only surface reaction. Importantly, the *in situ* ETS characterization of the PtNWs surface is naturally correlated with the in-device CV measurement. The ETS signal can be readily calibrated using the redox charges during the baseline conditions (see Supplementary Fig. 10), and then be used to quantitatively analyse the PtNW surface conditions under electrocatalytic reactions such as PROR. As shown in Fig. 4d, we have observed a drop of *G*_SD_ values with increasing amount of H_2_O_2_ at a given anodic potential. This trend can be used to estimate the surface coverage of O species at different reaction conditions, using the calibration obtained from the baseline conditions (no PROR) (see Supplementary Fig. 11). These analyses demonstrate that the ETS approach can be coupled with the traditional quantitative electrochemical analysis, and is capable of providing quantitative information during *in situ* monitoring of the electrochemical interfaces. More importantly, it can be continuously used to derive quantitative surface information that cannot be obtained from traditional CV measurement during practical electrocatalytic reactions.

### *In situ* ETS study of methanol and formic acid oxidation

**Figure 5:**
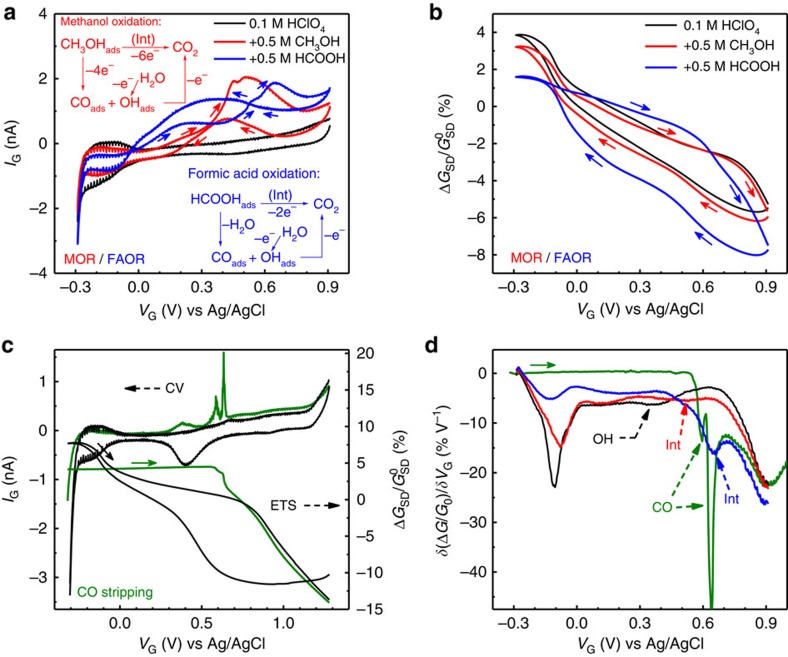
Methanol oxidation reaction (MOR) and formic acid oxidation reaction (FOR) on PtNWs. (**a**,**b**) *I*_G_*–V*_G_ (**a**) and normalized *G*_SD_*–V*_G_ (**b**) characteristics of a typical PtNW device during electro-oxidation of methanol (red curve) and formic acid (blue curve), as compared with the baseline process in HClO_4_ (black curve). Insets in **a** illustrate the reaction scheme of the ‘dual-pathway' mechanism for the electro-oxidation of methanol and formic acid on Pt catalyst. (**c**) *I*_G_*–V*_G_ (dashed curve) and corresponding *G*_SD_*–V*_G_ (solid curve) characteristics of PtNWs for CO striping (purple curve) and baseline measurements (black curve). (**d**) Differentiated ETS (DETS) curves of PtNWs during MOR (red), FOR (blue) and CO stripping (purple). Solid arrows in all figures indicate the potential sweeping direction.

We have further explored the ETS technique for more complicated and practically important systems. The oxidation of methanol and formic acid with a Pt-based catalyst is the most extensively studied model reactions due to their potentials as the logistical energy feedstock for fuel cell technologies^32^. Compared with PROR, methanol/formic acid oxidation reactions (MORs/FAORs) involve a more complicated surface process based on the adsorption of C_1_ molecules. By far, fundamental research on both reactions was mostly assisted by infrared-based spectroscopies. Although some exact details remain elusive, a ‘dual-pathway' mechanism (including (1) a direct oxidation via reactive intermediate and (2) an indirect oxidation via self-poisoning intermediate CO) has been established for both reactions (Fig. 5a; inset). We have used PtNW ETS to explore these reactions. *I*_G_–*V*_G_ showed typical CV characteristics of MOR/FAOR (Fig. 5a), with the electrical transport signal (Fig. 5b) obtained at the same time. Similar to the ETS studies in PROR, the indicative signals came from the change in ETS curves relative to the ‘baseline' curve, which was established with no reactant.

A noticeably lower *G*_SD_ value (compared with the baseline condition without methanol or formic acid) was observed in the H adsorption/desorption region (H_upd_) region for both MOR and FAOR (Fig. 5b, *G*_SD_ value at H_upd_ potential region: HClO_4_>CH_3_OH>HCOOH), which is consistent with the observed trend in each H_upd_ current in the CV results, indicating the H adsorption sites were partially occupied by methanol and formic acid (or unreacted intermediates) resulting a less complete coverage of H adsorption on Pt surface. At the oxidation potential range (>0 V versus Ag/AgCl), MOR resulted in little change in *G*_SD_ compared with the baseline curve (Fig. 5b, red versus black curve), whereas the shape of *G*_SD_ curve altered significantly in FAOR (Fig. 5b, blue curve). The obvious distinction in the ETS results between MOR and FAOR is interesting, as they share analogous reaction mechanisms and similar CV characteristics (Fig. 5a). Although the *in situ* infrared spectroscopy has been extensively used to study the surface chemistry and reaction kinetics for both Pt-catalysed MOR and FAOR, their direct comparison has been rarely conducted in one study. On the basis of time-resolved *in situ* infrared studies, a major difference between MOR and FAOR is the accumulation of surface ‘poisoning' intermediate (CO_ads_). For MOR, CO_ads_ starts to accumulate at onset potentials for oxidative methanol dehydrogenation: CH_3_OH→CO_ads_+4H^+^+4e^−^, at 0.10 V versus Ag/AgCl^33^, whereas for FAOR, CO_ads_ starts to accumulate at much more negative potentials due to the non-oxidative dehydration of formic acid: HCOOH_ads_→CO_ads_+H_2_O (a pre-covered 0.5 ML of CO_ads_ can be achieved at −0.15 V versus Ag/AgCl before FAOR at higher potentials)^34^,^35^. Moreover, the faster CO_ads_ accumulation in FAOR than in MOR has also been observed recently in *in situ* open circuit potential studies^36^. This stronger ‘self-poisoning' effect accounts for the strongly suppressed CV current during positive scan of FAOR, especially for reactive facet such as Pt(100)^32^,^37^,^38^. Importantly, the surface conditions of this ‘self-poisoning' process can be directly visualized in ETS signal: a slightly altered ETS was observed for a weakly poisoned Pt surface during MOR, while a heavily distorted ETS was observed for a strongly poisoned surface in FAOR (Fig. 5b).

To fully understand the different ETS characteristics in MOR/FAOR and their correlations with the CO_ads_ poisoning, we have carried out the ETS measurement in the CO stripping process (Fig. 5c, with differentiated ETS data shown in Fig. 5d). During the CO stripping measurement, the surface of PtNWs is first fully covered with a monolayer of CO_ads_ until it is oxidized in the positive potential sweeping. In general, one broad CO oxidation peak is expected at around 0.50–0.60 V versus Ag/AgCl in the positive CV scan for bulk PtNWs in a typical CO striping process. It is interesting to note that two sharp oxidation peaks were observed at 0.55 and 0.6 V versus Ag/AgCl, indicating distinct CO_ads_ sites with slightly different binding energies. The observation of multiple distinct peaks is probably because the device is composed of limited number of nanowires, therefore it is more likely to distinguish different binding sites that is difficult to resolve in bulk CV measurements. The ETS measurement shows a flat *G*_SD_ curve (Fig. 5c, green curve) until the CO_ads_ starts to be oxidized at the CO oxidation onset potential. This flat ETS feature is attributed to the stable CO_ads_ layer that blocks the adsorption (and therefore scattering) of other possible surface adsorbates until CO desorbs on oxidation. It should be noted that the flat CO stripping ETS curve lies below the baseline ETS at H_upd_ region and above the baseline ETS at DL region (Fig. 5c, green versus black curves). This result indicates the strength of surface scattering for three differently terminated Pt surfaces: Pt–H<Pt–CO<Pt–H_2_O. Comparing the ETS curves obtained from PtNWs during CO stripping, MOR and FAOR, it is obvious that the more distorted ETS characteristic for FAOR during positive potential scan, including a lower value of *G*_SD_ in H_upd_ region and a higher *G*_SD_ value in DL region, can be explained by partially covered CO_ads_ during this potentiodynamic FAOR process. At the beginning of the second oxidation peak for FAOR during positive CV scan (Fig. 5a), a sharp drop in *G*_SD_ signal is observed (Fig. 5b), with a corresponding peak in the DETS (Fig. 5d). It is important to note that the onset potentials for this sharp drop in *G*_SD_ coincides with the CO_ads_ oxidation potentials in the CO striping measurement (Fig. 5d), strongly suggesting that this drop in *G*_SD_ can be largely attributed to the desorption of CO_ads_ and adsorption of other molecules (H_2_O and O species) on the Pt surface. Overall, these studies demonstrate that ETS may offer a valuable signalling pathway for probing the surface poisoning level of metal catalysts, which can facilitate the design and development of high-performance electrocatalysts.

It is noticed that the other half of the FAOR ETS at the negative potential sweeping is also considerably different from baseline and MOR ETS, with the *G*_SD_ value considerably lower (Fig. 5b). The overall smaller *G*_SD_ value in the negative sweeping direction cannot be attributed to the CO_ads_, because the Pt surface starts from a CO_ads_-free condition^34^,^35^,^38^. Considering its significant difference from MOR ETS (with similar intermediate and reaction kinetics), this distinct feature in FAOR may be possibly attributed to specially adsorbed anions, HCOO^−^, which are not present in the baseline or MOR measurement. This explanation is consistent with a HCOO^−^ pathway for the mechanism of Pt-catalysed FAOR (that surface adsorbed HCOO^−^ is the active intermediate for the direct oxidation of FA, even at low pH conditions^38^), which has been recently proposed against other contradictory mechanisms^34^,^39^,^40^,^41^,^42^. Our ETS evidence suggests that specially adsorbed anions (HCOO^−^) may play an important role in the surface chemistry during FAOR, which favours the newly proposed HCOO^−^ mechanism.

## Discussion

In conclusion, we have developed a novel on-chip ETS approach for directly probing the electrochemical interfaces based on nanoelectronic signalling. The concurrent in-device voltammetry and *in situ* conductance measurement offers a complementary strategy to the traditional spectroscopy-based characterization techniques for *in situ* monitoring of the electrochemical surface conditions of metal nanocatalysts. It possesses unique advantages such as strictly surface-selective signals, no involvement of external radiation energy, miniaturized, scalable and CMOS compatible lab-on-a-chip set-up and low requirements on instrumentation. The intrinsic characteristics of electrical transport measurement could potentially lead to future methodologies for high-throughput measurements and small-scale sampling down to the single-particle level. It can thus enable a useful and convenient tool to promote fundamental understanding and future development of advanced nanoelectrocatalysts. Furthermore, the coupling of traditional electrochemical methods such as voltammetry offers the capability of extending this system to a wide range of interdisciplinary research field (that is, biological materials/systems) where electrochemical process plays a central role.

## Methods

### Synthesis of PtNWs

PtNWs were synthesized following a previous procedure^22^ with slight modification. Typically, a mixture of KOH (0.6 g) and ethylene glycol (4 ml) was dissolved in DMF (6 ml). Aqueous solution of K_2_PtCl_6_ (8 wt%, 0.1 ml) was then added into the mixture. After stirring for 20 min, the reaction mixture was transferred into a Teflon-lined autoclave, which was maintained at 150 °C for 15 h and then cooled to room temperature. The black powders were collected after the reaction and washed with ethanol and deionized (DI) water repeatedly for several times before use.

### Preparation of PtNWs films

A free standing film was assembled from as-prepared PtNWs suspension by a co-solvent evaporation method^23^. Typically, PtNWs suspensions in ethanol (400 μl, 0.4 mg ml^−1^) was mixed with DI water (miliQ filtered, 600 μl) and *n*-butanol (250 μl). The suspension of PtNWs in mixed solvents was added drop by drop into a flask (about 9 cm in diameter) filled with DI water. A film of PtNWs was then formed on the water surface and was later transferred onto the device.

### Fabrication of the PtNWs electrochemical device

Typically, a PMMA (A8, MicroChem Corp.) film was prepared by spin coating on the substrate (p++ silicon wafer with 300 nm thermal oxide) surface with pre-patterned Au electrodes (Ti/Au, 50/50 nm). E-beam lithography was then used to open windows on PMMA, which created desired patterns on the substrate. The pre-prepared (by co-solvent evaporation) free standing film of PtNWs was then deposited onto the substrate surface. After the removal of PMMA template, PtNWs was deposited on the device substrate with desired patterns. To eliminate the influence of electrolyte and to avoid electrochemical reactions on the metal electrodes, another layer of PMMA (∼500-nm thick, electrochemically inert) was then deposited on the PtNWs device with spin coating. A smaller window that only exposes PtNWs was opened by e-beam lithography. The final device, with exposed PtNWs and PMMA protected electrodes was used for in-device electrochemistry and *in situ* electrical transport spectroscopy measurement.

### In-device CV and *in situ* ETS

A two channel SMU (Agilent B2902a) was used for the measurement. The first SMU channel was used as a potentiostat to control the potential of source electrode as to the reference electrode (*V*_G_), while collecting the current (*I*_G_) through the counter electrode. In a typical in-device CV measurement, the scan rate is 32 mV s^−1^. The second SMU channel was used to supply a small potential (50 mV) between source and drain electrodes and collecting the corresponding current (*I*_SD_). For a typical measurement in this study, the gate/faradic current is generally several orders of magnitude smaller than the ETS current (*I*_G_ ∼1 nA and *I*_SD_ ∼10 μA). Therefore, the in-device CV current does not affect the ETS current and no additional background subtraction or other mathematical treatment is needed before the data analysis. A more detailed description of the measurement can be found in Supplementary Methods.

### Normalization of the *I*_SD_–*V*_G_ results

In an aqueous environment, the conduction electrons were at least scattered by surface adsorbed water molecules, the conductance of PtNWs at this stage is considered as ‘baseline' conductance (*G*_SD_^0^). This baseline value can be determined before each electrical spectroscopic scan by measuring the *I*–*V* characteristics of PtNWs with no *V*_G_ applied. With *G*_SD_^0^ measured, the *I*_SD_ of each test could also be normalized to relative conductance change (Δ*G*_SD_*/G*_SD_^0^, where *G*_SD_=*I*_SD_/*V*_SD_ and Δ*G*_SD_=*G*_SD_−*G*_SD_^0^). This normalization does not change the characteristic of each *I*_SD_*–V*_G_ curve, and makes the comparison between different scans and different devices more reasonable, as the baseline conductance of each device are different and each could drift during measurements, due to the reasons such as Pt atom dissolution. The shape of *I*_SD_, *G*_SD_ and Δ*G*_SD_ are the same, and in the paper this characteristic is all referred as *G*_SD_ result.

## Additional information

**How to cite this article:** Ding, M. *et al*. An on-chip electrical transport spectroscopy approach for *in situ* monitoring electrochemical interfaces. *Nat. Commun.* 6:7867 doi: 10.1038/ncomms8867 (2015).

## Supplementary Material

Supplementary Information Supplementary Figures 1-11, Supplementary Discussion, Supplementary Methods and Supplementary References
