# Supplementary material for: An on-chip electrical transport spectroscopy approach for in situ monitoring electrochemical interfaces
Source: Nat Commun. 2015 Aug 6;6:7867. doi: 10.1038/ncomms8867 (PMC4918314; doi:10.1038/ncomms8867)
Supplement: Supplementary Information — Supplementary Figures 1-11, Supplementary Discussion, Supplementary Methods and Supplementary References [file ncomms8867-s1.pdf]

1. Supplementary Figures

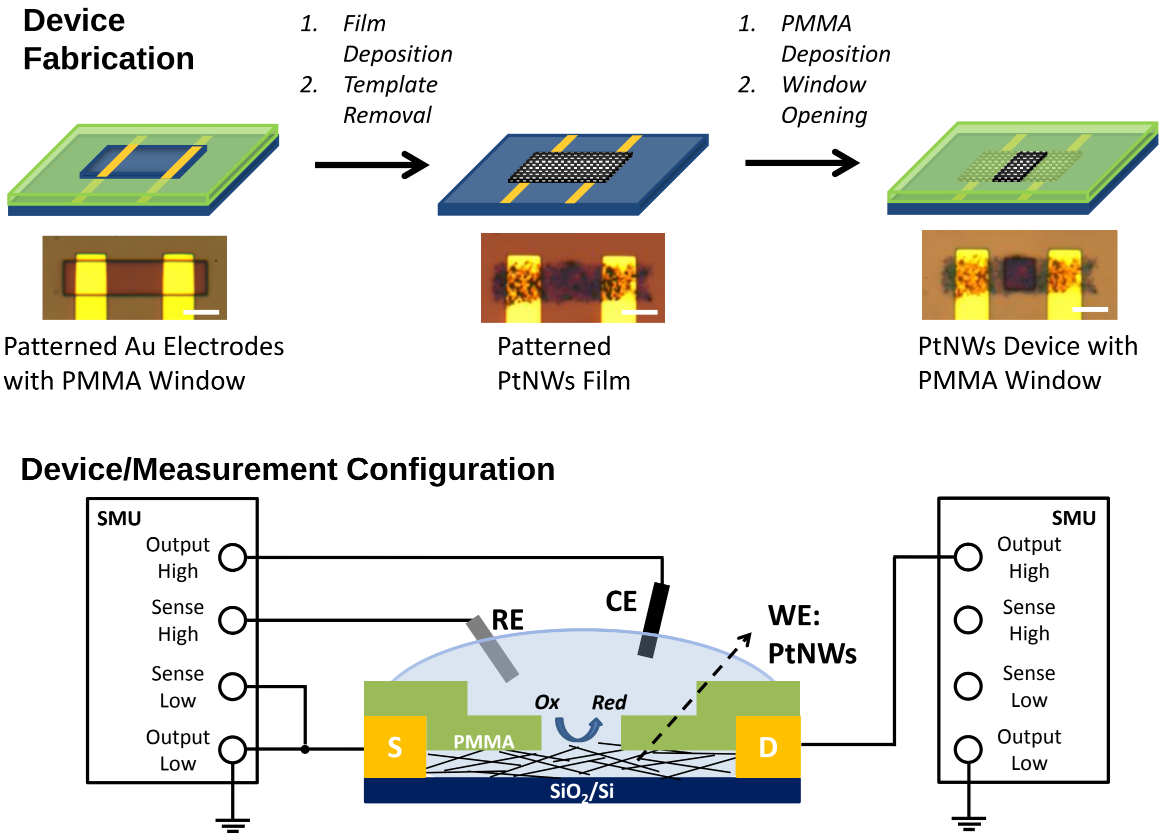

Supplementary Fig. 1| Schematic illustration and optical images of device fabrication (top) and schematic illustration of the cross-sectional view of the device and the measurement configuration (bottom). Scale bars in the optical images are 5 μm.

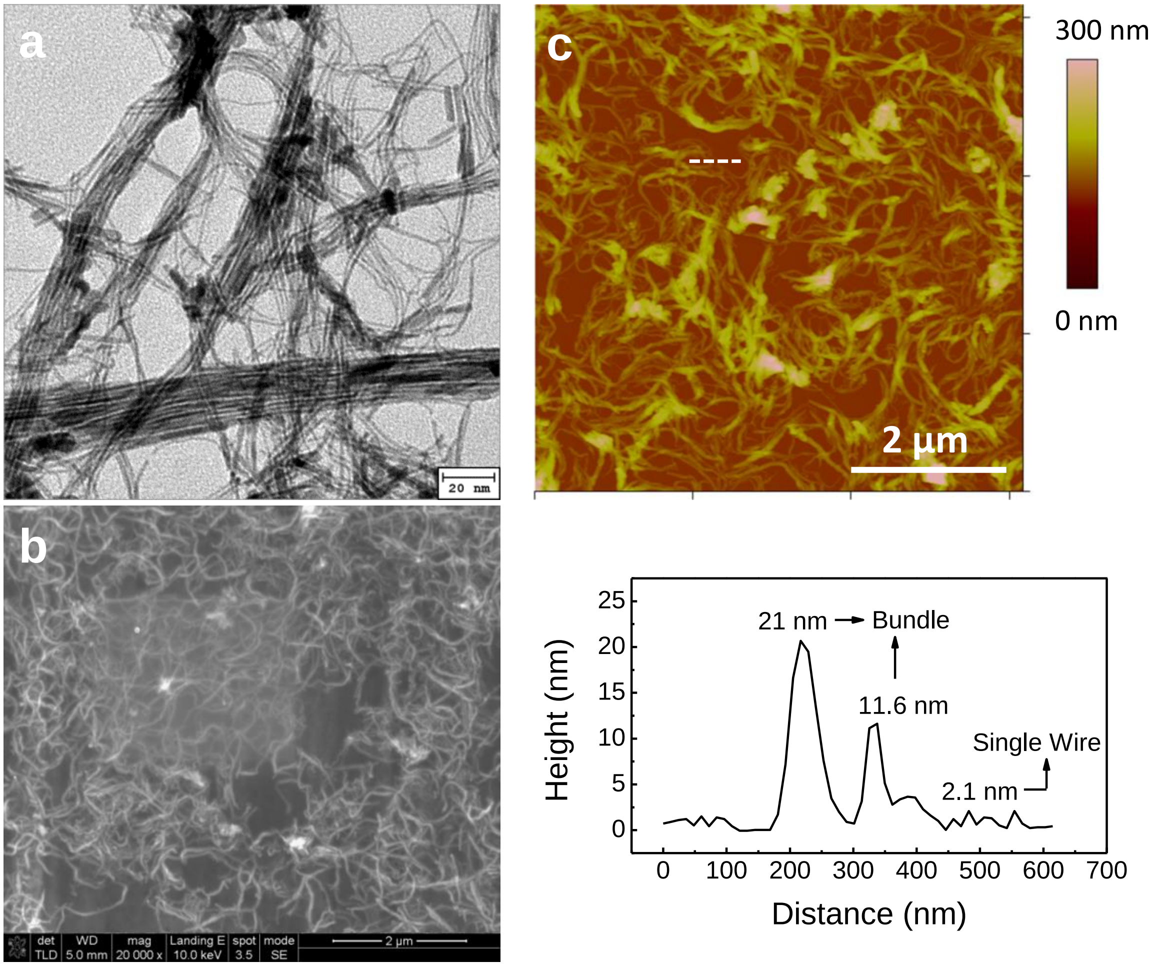

Supplementary Fig. 2| Representative TEM (**a**) and SEM (**b**) images of as-prepared PtNWs, and AFM (**c**) image of PtNWs film on a device. TEM and AFM section height profiles (bottom) show that PtNWs exist mostly as bundles in the network.

In situ ETS during Electrochemical Cleaning

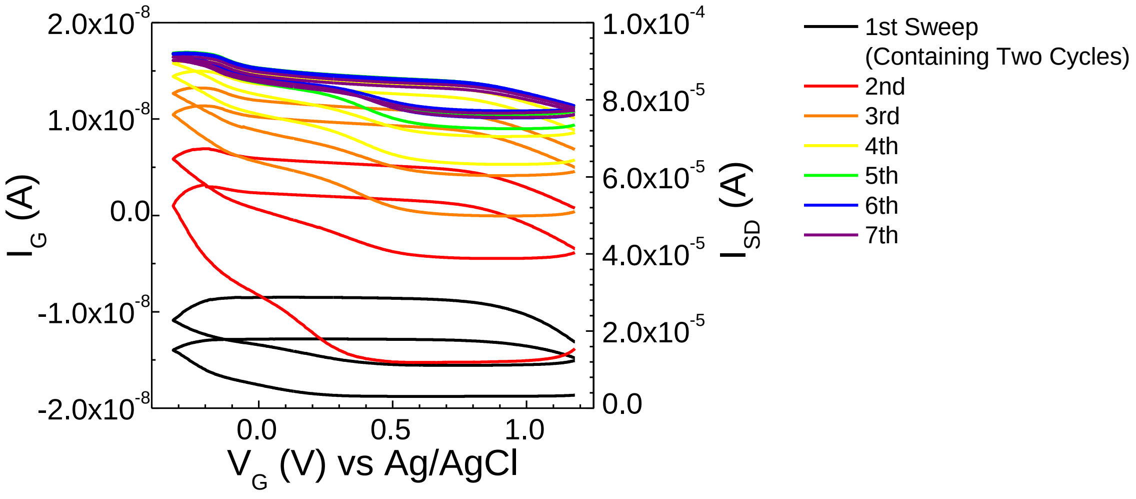

Supplementary Fig. 3| I_{SD}-V_G characteristics of PtNWs recorded during the first 7 sweeps (each sweep contains two potential cycles) of a PtNWs device before the standard in-device CV and in situ ETS measurement.

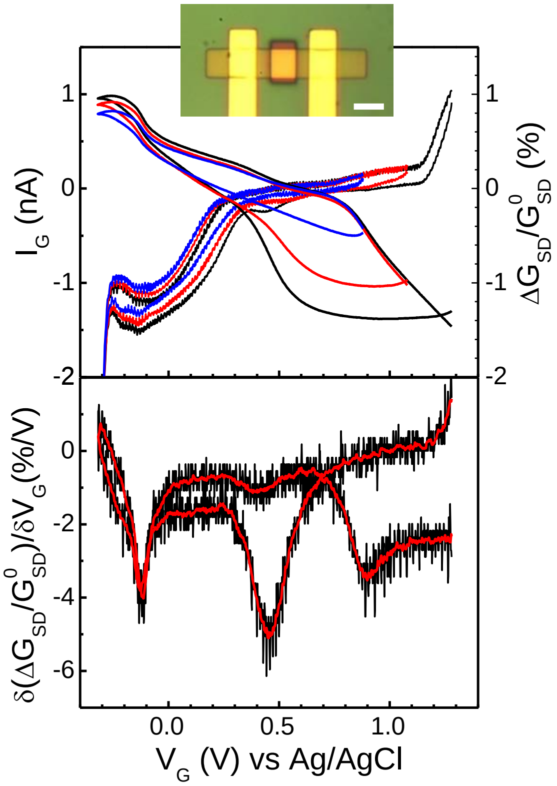

Supplementary Fig. 4| a, I_G-V_G (CV) and G_{SD}-V_G (ETS) characteristics of a Pt thin film prepared by the deposition of evaporated Pt. Inset depicts an optical image of such Pt thin film device, which has the same 5μm x 10μm dimension as PtNWs device used in this work and 10nm in thickness. Scale bar is 5μm. **b,** Differentiated ETS curve of Pt thin film.

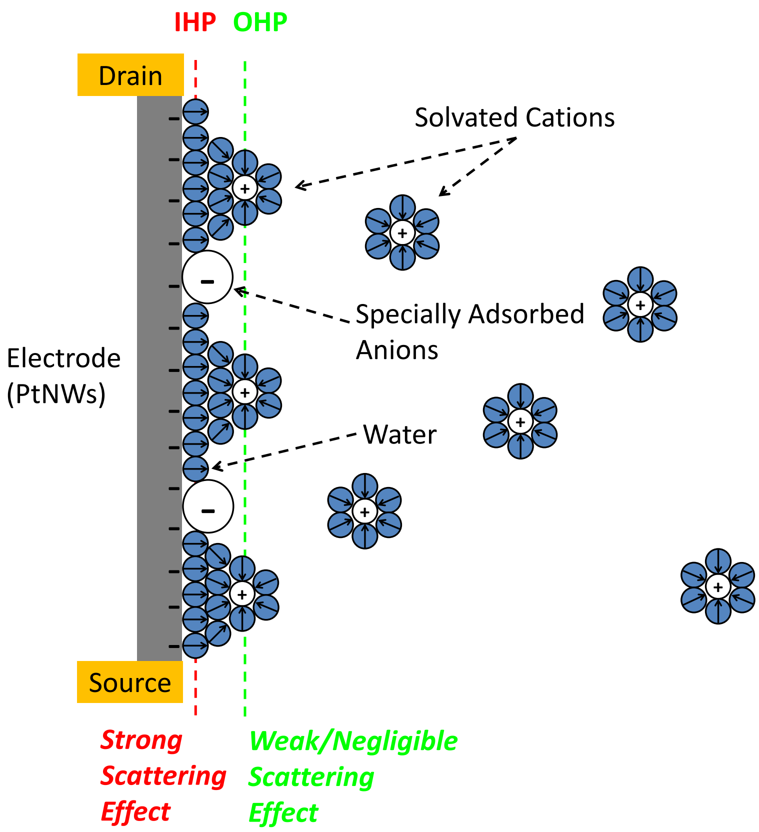

Supplementary Fig. 5| Schematic illustration of a double layer model in the measurement of in-device CV and in situ ETS measurement. The inner Helmholtz plane (IHP) is defined by a monolayer of adsorbed solvent molecules and specially adsorbed anions (do not exist in 0.1M $HClO_4$ electrolyte). This IHP results in the strong surface scattering effect that determines the ETS signals.

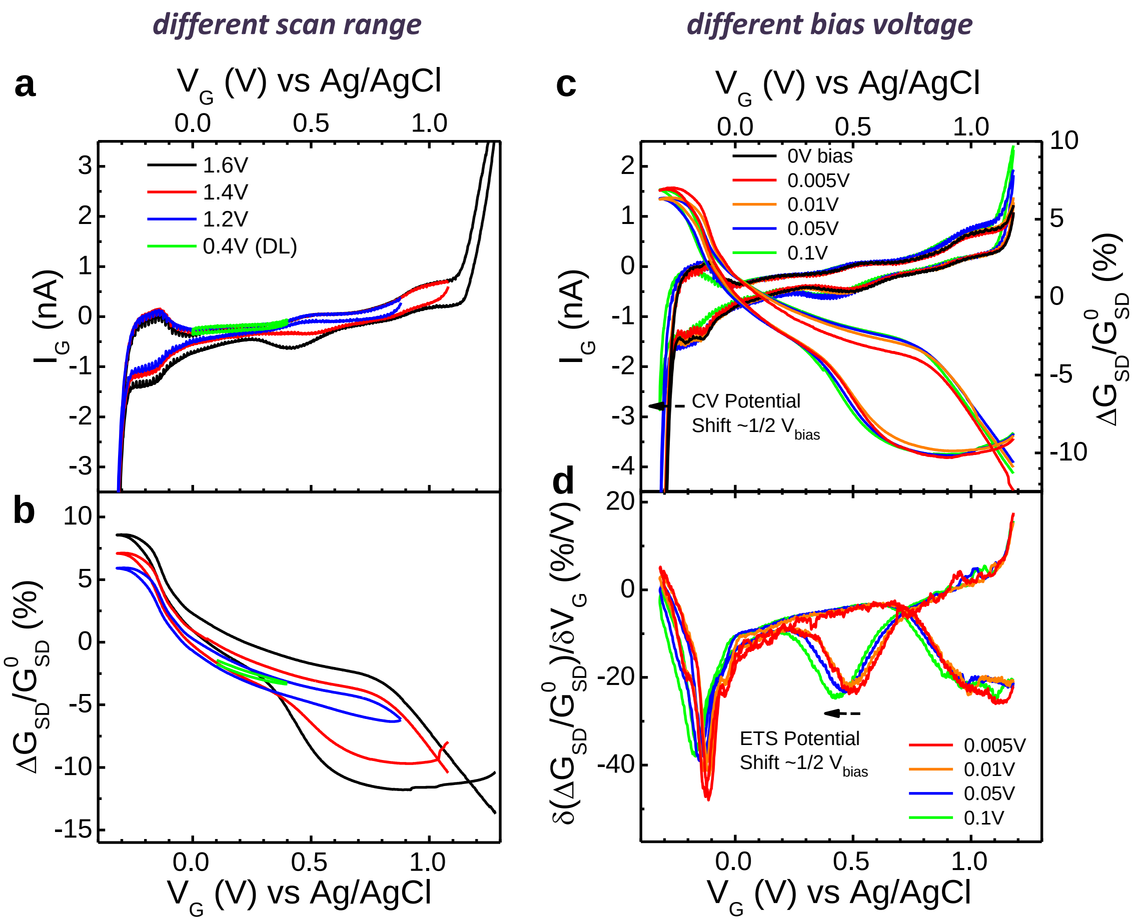

Supplementary Fig. 6| a,b, I_G-V_G(**a**) and G_{SD}-V_G(**b**) characteristics of PtNWs with different potential scan range, including within only double layer charging region. **c,d,** I_G-V_G, G_{SD}-V_G(**c**) and differentiated G_{SD} (**d**) characteristics with different bias voltages. Both results show a potential shift that is roughly about half of the V_{bias}.

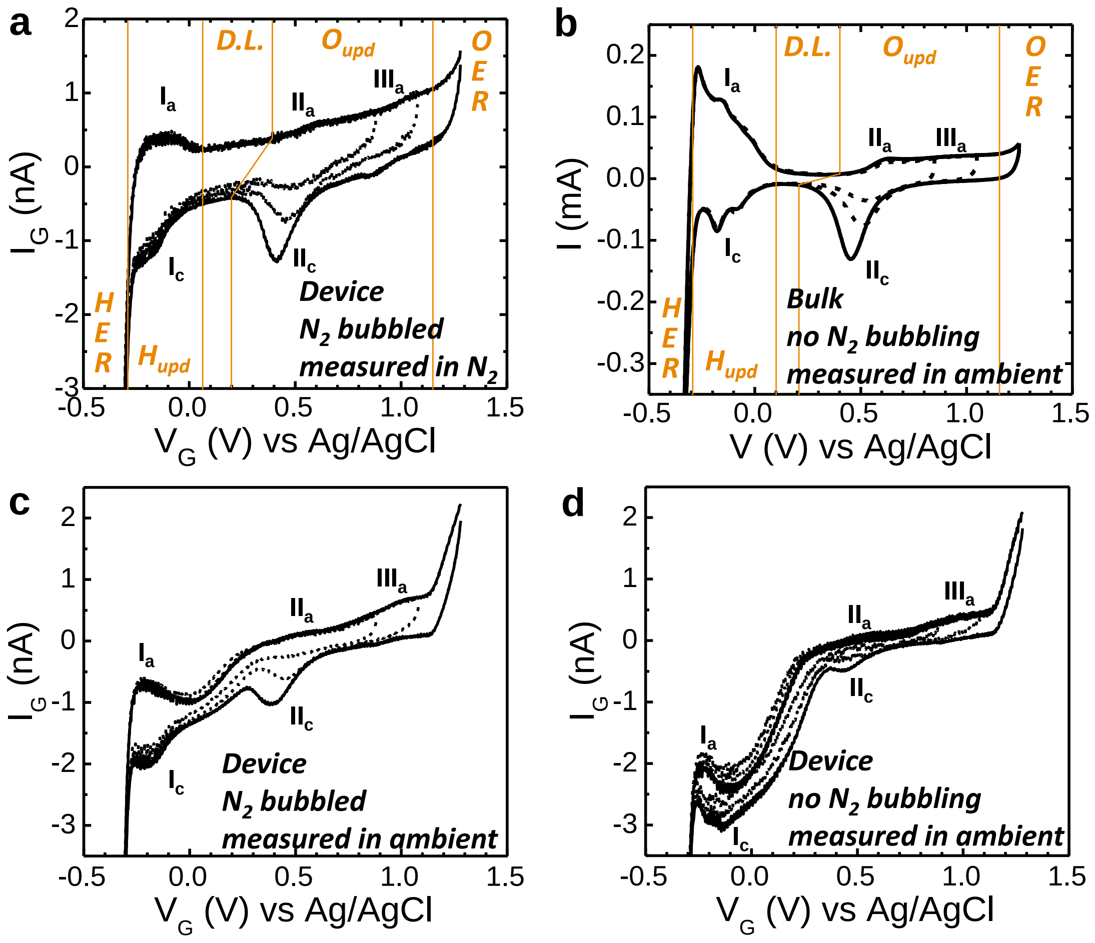

Supplementary Fig. 7| Typical cyclic voltammogram of PtNW device measured by in-device voltammetry (SMU, Agilent) (**a, c, d**) and PtNWs drop-casted on the glass carbon electrode measured by standard potentiostat (VersaStat, Princeton) (**b**). CV result in **a** was measured with N_2 bubbled electrolyte and in N_2 environment, result in **b** and **d** were measured with as-prepared electrolyte and inert environment, result in **c** was measured with N_2 bubbled electrolyte and in ambient environment. Five potential regions were identified on all voltammogram: hydrogen evolution reaction (HER) region; hydrogen adsorption/desorption region (H_{upd}); double layer region (D.L.,); surface oxide formation/reduction region (O_{upd}); and oxygen evolution reaction (OER) region. Two cathodic and three anodic processes (peaks) were observed: hydrogen adsorption (I_c); oxide reduction/desorption (II_c); hydrogen desorption (I_a); hydroxyl adsorption (II_a); and surface oxide formation (III_a). In all voltammogram, electrolyte is 0.1M $HClO_4$, potential scan rate is 32 mV/s, and N_2 is ultrahigh purity grade (Air Liquide).

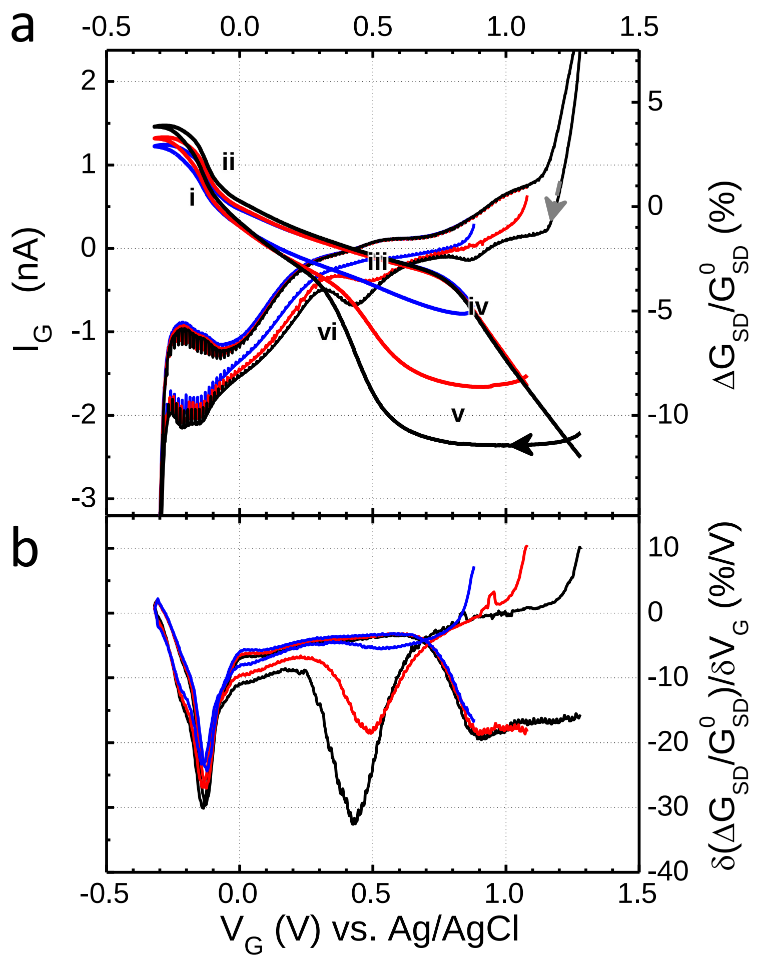

Supplementary Fig. 8| a, Typical I_G-V_G and corresponding G_{SD}-V_G characteristics of a PtNWs device with different potential sweeping ranges (upper potential limit). **b**, corresponding differentiated G_{SD} characteristics.

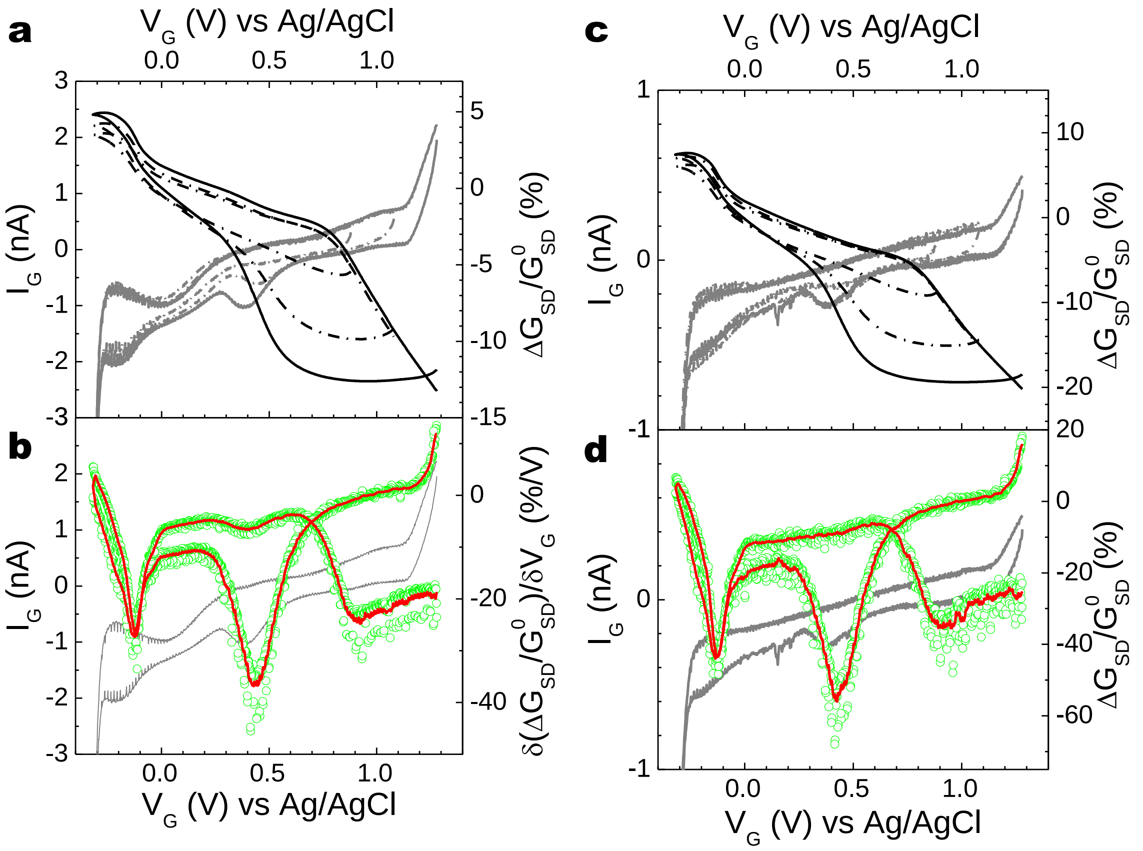

Supplementary Fig. 9| Typical I_G-V_G and G_{SD}-V_G and electrical spectroscopy data of two PtNWs devices with different OH_{ads} signals.

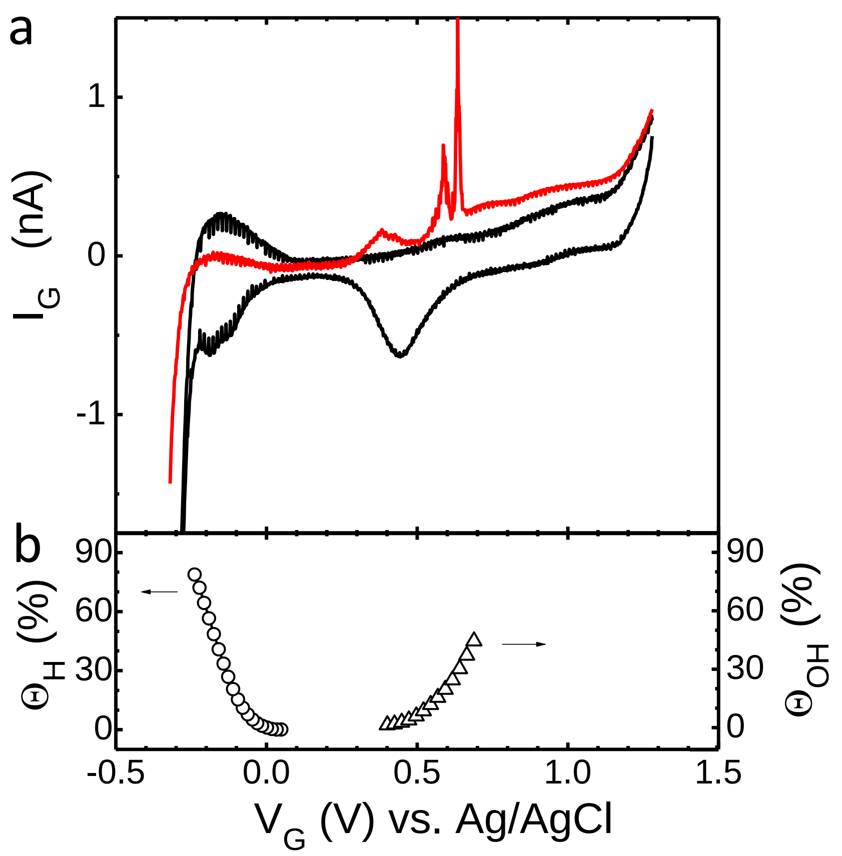

Supplementary Fig. 10| CO striping measurement of PtNWs. a, I_G-V_G characteristics of a PtNWs device for CO stripping (red) and baseline in N_2 bubbled 0.1M $HClO_4$ (black). **b**, Surface coverage of H_{ads} in H_{upd} region and OH_{ads} in O_{upd} region, calculated from integrated charge in comparison with CO stripping charge measured.

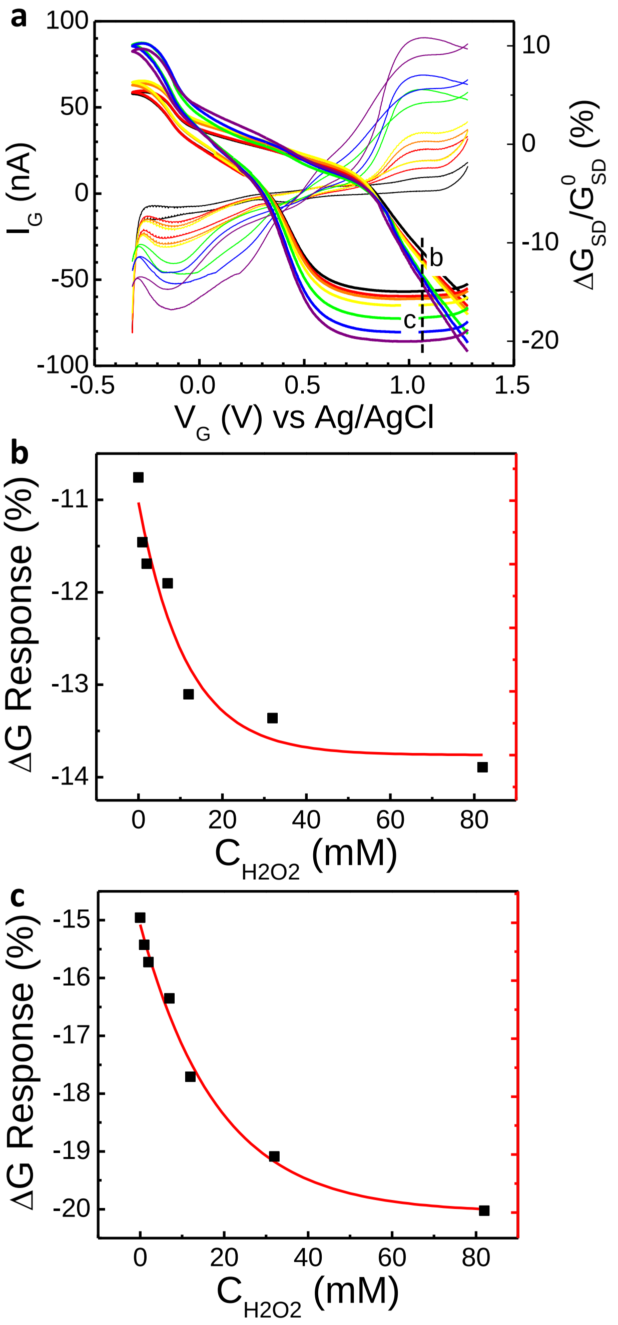

Supplementary Fig. 11| I_G-V_G and G_{SD}-V_G characteristics of three PtNWs device during PROR (**a**) and respective quantitative relationship between G_{SD} response and H_2O_2 concentrations (**b, c**). Red curves in (**b, c**) is first order exponential fitting of the G_{SD} value (black square) at the cathodic current peak potential (indicated by the black dashed line) under different H_2O_2 concentrations.

2. Supplementary Discussion

2.1 Theoretical consideration of size effect and surface scattering induced response of ultra-fine metal nanowires. Based on the mathematical model developed by Dingle to describe the conductivity of one-dimensional (1D) cylindrical metal wires, the dependence of metal wire resistivity on their diameter/mean free path has been summarized in literature[2]. The resistivity of metal wire divided by the resistivity of bulk metal is plotted to the ratio of diameter over electron mean free path, as shown in Fig. 1 in the main text. This relationship summarizes the size effect on the electrical transport property of the metal thin wires.

Moreover, upon surface adsorption of gas molecules, the resistivity of metal thin wire is changed due to the additional surface scattering of electrons by the adsorbed molecules. Since these additional scattering centers are strictly on the surface, the adsorbed molecules can be viewed as only to induce the change of p value (portion of specular scattering vs. diffusive scattering on the surface), while keeping the λ value (electron mean free path corresponding to the metal wire material) constant. Therefore, the theoretical response can be estimated by calculating the resistivity change from the change of p values. For example, theoretical maximum response that can be induced to a given metal thin wire due to the molecular adsorption induced surface diffusive scattering can be calculated for the change of p=1 to p=0. The calculated response, $\Delta\sigma/\sigma_0$, is plotted as down triangles in Fig. 1 in main text. For a moderate case where p changes from 1/2 to 0 upon surface adsorption, the response is calculated as $\Delta\sigma/\sigma(p=1/2)$, plotted as up triangles in Fig. 1 in main text as well. We can conclude that both size effect and surface scattering induced response in metal wire resistivity increase dramatically with reduced diameter of wires, especially when it approaches to the corresponding mean free path of the metal wires. This gives the fundamental basis for our nanoelectronic signaling pathway, which measures the electrical transport properties of ultra-fine metal nanowire to reveal its surface conditions during a complex electrochemical process.

2.2 Theoretical consideration of the physical origin of the ETS signals (G_{SD} curve) of PtNWs. The interpretation of the *in situ* ETS results during any electrochemical processes is based on an important assumption that the G_{SD} (ETS) signals are exclusively (or at least mostly) determined by the change of surface conditions (i.e., from surface scattering as discussed in the above section). Due to the possibility of structural variation of the PtNWs material and complicated environment change that is involved in any electrochemically process, conclusive identification of the physical origin requires carefully exploration. We have designed and conducted a series of control experiments, which collectively rule out the possibility of other sources and link the ETS signals exclusively to the surface scattering effect from the surface adsorbed chemical species (which defines the inner Helmholtz plane in a double layer model).

2.3.1 Surface Structural Variations.

Electrochemical pre-cleaning of the device before standard CV and ETS measurements. For the CV and *in situ* ETS data presented in our manuscript, the surface of PtNWs has been pre-cleaned electrochemically to a stable state by multiple cycles of CV before the ETS test, following the typical electrochemical cleaning procedures used in standard CV measurements.

The use of multiple cyclic potential sweeps can remove possible surface contaminants and generate a relatively stable electrode surface (after possible surface rearrangement) for the reliable and repeatable CV results. Similarly, the ETS signals we have shown in the manuscript are the results obtained after the stable surface has been generated by electrochemical cleaning. After this step, both CV and ETS characteristics of the PtNWs are stable and reproducible. We further show the result of ETS signals during such electrochemical cleaning process (Supplementary Fig. 3), which demonstrates the signal for a process where surface structure of PtNWs is irreversibly changed. As shown in supplementary Fig. 3, the I_{SD} demonstrates a significant and continuous increase (up to one order of magnitude) during the first several cycles of potential sweeps. This can be rationalized by the removal of surface contaminations (and the associated surface scattering effect) and significantly improved wire to wire contact (due to a cleaner surface or tighter attachment) during the surface cleaning process. After ~15 cycles, the ETS signal presents a stable and reproducible characteristic (as the one shown in Fig. 3a in main text).

As well-established in Pt electrochemistry, a stable and reproducible CV profile of a Pt electrode (in $HClO_4$ electrolyte) represents its intrinsic characteristic for the surface electrochemical processes[3]. Therefore, after achieving the same stable and reproducible CV profiles of PtNWs, the stable and reproducible ETS profile obtained correspondingly (both shown in Fig. 3a in main text) is also believed to be intrinsic to such surface electrochemical processes. One can argue that certain surface variations may still exist during such electrochemical processes (for example, at a highly positive potential, surface adsorbed O species start to penetrate underneath the surface Pt atoms, which may be viewed as a surface structural variation). These types of surface variation are reversible during the electrochemical cycles, and can be reflected in the reproducible ETS characteristics. Therefore, they are fundamentally different from those irreversible surface structural variations as shown in supplementary Fig. 3. Eventually, the former reversible surface structural variations can be consistently reflected in all ETS related studies, and can be viewed as a specific type of electrochemical process.

In-device CV and *in situ* ETS characteristics of a Pt thin film fabricated from deposition of evaporated metal. Supplementary Fig. 4 demonstrates the ETS characteristics of a Pt thin film (with same 5μm x 10μm dimension and 10nm in thickness) under the same test conditions as PtNWs. The Pt thin film is fabricated through a different approach: e-beam evaporation of the Pt metals. For the Pt thin film, the G_{SD} curve demonstrates the same qualitative characteristic as PtNWs (shown in Fig.3a in main text), but with a much lower response value. Since the Pt thin film is prepared with a complete different fabrication method and with different surface morphology (e.g., surface roughness and grain boundary), this result demonstrates that the ETS characteristic we observed in PtNWs is not affected by the starting surface morphology of Pt material, but rather be intrinsic to the Pt material with specific electrochemical properties.

In summary, our results (Supplementary Fig. 3 and Fig. 4) demonstrate the possible I_{SD} variations that could be observed in case of different surface structural variations (surface rearrangement, surface/interface cleaning, different surface morphologies such as surface roughening and grain boundary). Overall most of these surface structural changes are unlikely to be reversible, and therefore the reproducible CV and ETS characteristics obtained during the repeated potential cycles do not reflect any of these irreversible structural changes. The electrochemically induced reversible structural variations may exist, but such variations are intrinsic to a given electrochemical process and are also well reflected in the ETS signals.

2.3.2 Environmental Variations.

Another possible factor that could influence the ETS signals is the environmental change during ETS measurement. The environment for PtNWs during an electrochemical process (in the presence of solvent, electrolyte and charges induced by electrochemical potential) is much more complicated than those considered in ambient/gas or vacuum environment. To better discuss such complex environment, a double layer model (which is used for general electrochemical or electrolyte-gating discussion) is considered here. A schematic illustration of the double layer model for in-device CV and in situ ETS measurements is shown in supplementary Fig. 5. As clearly demonstrated in the double layer model, a closely adsorbed solvent molecules (without chemically bonded species at specific potentials or specifically adsorbed anions in 0.1M $HClO_4$, it should be H_2O_{ads}) that is in direct contact with the PtNWs surface defines an inner Helmholtz plane (IHP). The variation of these directly adsorbed molecules in IHP is considered as the major source of surface scattering based signals. Besides, two environmental changes that should be taken into account in the consideration of G_{SD} signals are: (i) the change of gating potentials (electrical field) that is applied for electrochemical reaction (the influence of the gating field effect on the device conductance is commonly seen in the semiconducting materials but not in metallic materials); (ii) the counter-charged ions (solvated) that is moving towards the surface of PtNWs (to as close as outer Helmholtz plane, OHP) when it is charged by applying electrochemical potentials. This could also be viewed as the change of double layer structures.

It should be firstly pointed out that one significant difference between above mentioned environmental factors (change of gating potential/field and counter ions/double layer structure) and the surface adsorptions (scattering) factors is that the environmental factors change continuously with the sweeping potential, while the surface adsorption factors do not change continuously according to the gate potential but according to the occurrence of specific chemical reactions. With this consideration, we have first conducted a control CV/ETS measurement with PtNWs at a potential range that only involves the double layer region (no other redox reactions). As shown in the Supplementary Fig. 6a and b, when the gate potential is limited to the double layer region where no electrochemical reactions take place, the G_{SD} does not demonstrate a significant variation. Instead, G_{SD} signals only become significant during the electrochemical reactions (in this case H_{upd} and O_{upd}). Since the environmental factors (potential/electric field and counter ions/double layer structure) keep changing at the double layer region, the negligible G_{SD} variation at this region can serve as solid evidence that the dominant G_{SD} signals from ETS measurements at the whole electrochemical potential region are originated from the potential specific surface absorption rather than the changes in environmental factors (that is not potential specific, but linear vs. potential).

Another more conclusive result is obtained in the CO stripping measurement, as demonstrated in the main text (Fig. 5c and d in main text). We have induced a layer of strongly adsorbed molecules (CO) on the surface of PtNWs (which will sustain a wide electrochemical potential range) and conducted in-device CV and *in situ* ETS measurement. According to the above summarized assumptions, the lack of electrochemically induced surface adsorption will generate a featureless G_{SD} signals in a wider range of applied gate potentials, despite the continuous change of environmental factors with sweeping potential. Indeed, during the CO stripping measurement, we have observed a flat G_{SD} curve from the very negative electrochemical potential (HER) up until a high positive potentials where CO_{ads} starts to desorb through its

oxidation, as shown in Fig. 5c and d in the main text. This result clearly demonstrates when the surface of PtNWs is passivated with a layer of strong binding adsorbates, the G_{SD} signal is not affected even if the environmental factors (potential/electric field and counter ions/double layer structure) is altered. Only when adsorbate desorbs due to electrochemical reactions, can we observe an obvious G_{SD} signal. Except for CO_{ads}, some other strong binding chemical species, for example, a specially adsorbed anions, I^-, can cause the same effect in a potential range when surface adsorbed I^- is not oxidized (the result with I^- binding is not included in this paper). These studies clearly demonstrate the ETS signal is surface specific, and not environmental specific (i.e. only depends on surface condition, but not on environmental change).

Besides the gate potential, a source-drain voltage has been applied for the G_{SD} measurement, which could also alter the electric field at the proximity of PtNWs. For the influence of V_{SD} on the electrochemical processes, it should be noted that the V_{SD} in our standard measurement is 0.05 V, which is relatively small compared with applied V_G up to 1.6V. Moreover, considering the thickness of the double layer (~1nm at 0.1M $HClO_4$) is much smaller than the channel length of PtNWs network (10μm), the V_{SD} is designed to have minimal influence on the double layer electric field and hence the electrochemical processes including transport of electrolytes and electrochemical reactions. To experimentally prove that applied bias voltage does not affect the in-device electrochemistry, we have carried out in-device CV and ETS measurement with PtNWs at different bias voltages of 0 V, 0.005 V, 0.01 V, 0.05 V and 0.1 V, as shown in Supplementary Fig. 6c and d. The CV characteristics of PtNWs does not change with the applied bias voltage up to 0.1 V, which means the electrolyte transport and electrochemical reaction are not significantly influenced by small V_{SD}. Instead, an overall potential shift was observed for CV results at increasing V_{SD}. Using HER potential as a reference point, the CV potential shift has been determined to be about half the value of the applied V_{SD}. This is not difficult to understand as the electrochemical potential (V_G) is actually applied between counter electrode and the source electrode (which is grounded). Therefore, for each point of PtNWs, depending on its distance to the drain electrode (d), it has an offset potential of $V_{SD}*d/d_{channel}$. The overall offset of the potential for the whole PtNWs film will then be ~ 1/2 V_{SD}. The corresponding ETS demonstrate a same shift, which is more obvious in the differentiated results (supplementary Fig. 6d). This observation means that although a small shift of electrochemical potential has been caused by applied V_{SD}, this does not influence the electrochemical process and *in situ* surface information obtained from the in-device CV and in situ ETS results.

In summary, the above mentioned control experiments collectively prove that the ETS signal of PtNWs is mostly originated from the surface adsorbed molecules, which is highly dependent on the surface electrochemical processes, and possibly from reversible surface variations that are also induced electrochemical processes. Other factors, including irreversible surface structural variation and environmental changes (potential/electric field and counter ions/double layer structure), do not significantly influence the ETS signal.

2.4 Electrochemistry of PtNW device and material. Supplementary Fig. 7 demonstrates the typical CV characteristics of PtNW device for *in situ* ETS studies (Supplementary Fig. 7a) and as-prepared PtNW material (Supplementary Fig. 7b). Five potential regions are presented in the CV characteristic of PtNWs with two cathodic and three anodic peaks.

The CV voltammogram showed a relatively small current when the potential was scanned into the double layer (D.L.) charging region (between 0.1 to 0.4V vs Ag/AgCl). As the potential sweep continued towards the negative direction (between ~0.1 to -0.3V vs Ag/AgCl), a monolayer of H atoms was adsorbed on the PtNWs surface prior to the evolution of H_2, and desorption of the surface H occurred after the scan reversed. This adsorption and desorption process resulted in a pair of reversible redox peaks I_c and I_a on the CV voltammogram respectively, and is defined as under potential H deposition region (H_{upd}). At the most negative potential (below -0.3V vs Ag/AgCl, a sharp increase in the cathodic current was observed, due to the hydrogen evolution reaction (HER).

The adsorption of oxygenated species began with the positive ongoing potential (above 0.4V vs Ag/AgCl), with a broad and flat anodic process observed in the CV characteristic. Two peaks were identified in CV, the first peak (II_a, around 0.6V vs Ag/AgCl) is assigned to the adsorption of hydroxyl species (OH_{ads}) and the subsequent peak at higher potential (III_a, around 1.0V vs Ag/AgCl) is assigned to the formation of adsorbed O atoms (surface oxide layer). At the reverse potential scan (from positive to negative direction), the reduction of oxygenated species was indicated by a cathodic current peak (II_c, around 0.45V vs Ag/AgCl) observed in CV. Such oxidation/reduction process (II_a, III_a, and II_c) is defined as under potential O deposition (O_{upd}) region. At the most positive potential (above 1.2V vs Ag/AgCl, a larger anodic current was observed, due to the oxygen evolution reaction (OER).

From the comparison between Supplementary Fig. 7a and Fig. 7b, the I_G-V_G curve obtained from a PtNW device closely resembles the CV characteristic of PtNW materials, with similar potential regions and cathodic/anodic peaks. This result demonstrates the validity of the in-device voltammetry using our measurement configuration. Several minor distinctions between the CV results from two methods are noticed. First, the H adsorption/desorption peaks from in-device CV curve lack the delicate feature compared with CV of bulk materials. This difference is due to the relatively small redox current (nA vs. mA) from the in-device electrochemical cell and micro-sized working electrode, which results in a small signal to noise ratio. Second, the in-device CV curve is a little “tilted” in shape. This is probably due to the trace amount of oxygen dissolved in the electrolyte solution. Compared with bulk electrode, our micro electrochemical cell is more sensitive to the trace oxygen, probably due to the micro structured working electrode and the dimension of the in-device electrochemical cell. As a result, the corresponding oxygen reduction current contributes to the “tilted” CV. CV curve shown in Supplementary Fig. 7a was obtained in a concealed box filled with inert gas, with electrolyte solution bubbled for at least 30 min inside the inert gas environment. For electrolyte without degas treatment or inert gas experiment environment, a more obvious “tilted” CV results were observed, as shown in Supplementary Fig. 7c and d.

2.5 Discussion on the upper potential limit dependence of CV and G_{SD} characteristics.

Electrochemical irreversibility has been commonly reported for the oxidation and reduction of Pt electrode at positive potentials, with asymmetric redox peaks in its typical CV characteristics [3]. Such irreversibility highly depends on the upper limit of the positive sweeping potential during CV, and was generally explained by the penetration of the oxygenated species under the surface Pt atoms, which would require lower potential for its reduction [3]. The dependence of irreversibility on the positive sweeping range was indeed observed on the in-device CV curve (I_G-V_G, Supplementary Fig. 8). More interestingly, similar potential dependence was observed on

G_{SD} as well ($\Delta G_{SD}/G_{SD}^0$-V_G, Supplementary Fig. 8), where the hysteresis of the curve matched the trend of upper potential limit and the voltammetric irreversibility. The hysteresis characteristics mainly came from the constant drop of G_{SD} value with the increasing potential. This observation indicated that the oxide formation process (which might include the O penetration) proceeded continuously, even after the beginning of OER. As discussed in the main text, the surface oxide formation (or penetration of O species) probably did not reach the maximum coverage after the onset of OER and was continued during oxygen evolution. In sum, the hysteresis of the G_{SD} curve was correlated to the electrochemical irreversibility of the nanomaterials, which i) offered nano-electronic proof for the molecular level explanation of the voltammetric irrversibility, and ii) provided an alternative indicative characteristic for such process besides the shape of their cyclic voltammograms.

2.6 Discussion on the OH_{ads} signal. Variations on OH_{ads} signals from different PtNWs devices were observed, some showed negligible change and some showed a small step of decrease in G_{SD}. Supplementary Fig. 9 demonstrates one of each typical situation. Overall, the relatively small signals from OH_{ads} can be attributed to the similar scattering effect between H_2O_{ads} and OH_{ads}, as they have similar orientations on the Pt surface through hydrogen bonding with neighboring adsorbates [4]. Previous theoretical and experimental data also indicated the existence of co-adsorption during their transition [5,6]. Variations of initial OH coverages on the as-prepared materials could also determine the intensity of the OH_{ads} signals, as the ultrathin nanowires were synthesized from a basic solution [1], we suspect that certain amount of hydroxyl groups were already formed on the as-prepared PtNWs. For higher pre-adsorbed hydroxyl coverage, PtNWs will demonstrate smaller signal for OH_{ads} and vice versa.

2.7 Quantitative consideration of G_{SD} characteristics for PROR. As discussed in the main text, the characteristics of G_{SD} and electrical transport spectroscopy of PtNWs provided tremendous information on the catalyst surface chemistry during a specific redox reaction such as PROR. As an electrical measurement, quantitative analysis also become possible for this technique that could potentially offer deep fundamental insights to the study, though measurement has to be handled with much more care, while precision, accuracy and reliability of the data is strictly required. Supplementary Fig. 11 demonstrated the I_G-V_G and G_{SD} measurement of three individual devices during PROR process, with varying H_2O_2 concentrations. It has been concluded in the main text that a larger fraction of the oxidized Pt surface or a larger coverage of O_{ads} was induced by further oxidization due to the presence of H_2O_2, which was indicated by the continuous drop of G_{SD} characteristics with the increasing H_2O_2 concentration. Multiple separate devices showed the same result for PROR, with G_{SD} at high potential range (cathodic current of PROR in CV) dropped to a lower value with increasing concentration of H_2O_2, with one example shown in Supplementary Fig. 11a. For further quantitative analysis, each G_{SD} value at the cathodic current peak potential (in CV) was plotted to the H_2O_2 concentrations. As can be seen in Supplementary Fig. 11b and c, the G_{SD} response followed an exponential dependence on the concentrations of H_2O_2. As the decay was resulted from the additional O coverage induced by adding the H_2O_2, the exponential fitting indicated a theoretical maximum surface coverage that could be resulted from the addition of H_2O_2. Combined with the calculated OH_{ads} coverage at baseline conditions (~45% calculated from our results as shown in Supplementary Fig. 10, as a reference, the O coverage derived from surface CV characteristics of a Pt(111) surface is ~45%

at 0.7V vs Ag/AgCl[7]), we can derived the relative surface coverage of O species at each H_2O_2 concentrations. This is a simple example of how electrical transport spectroscopy data can lead to quantitatively surface analysis of a studied reaction system. With future establishment of more reference points, the application of this electrical measurement technique could be greatly extended in the study of electrocatalytic reactions and catalytic materials, with fundamental insights derived from the quantitative analytical methods.

3. Supplementary Methods

Synthesis of PtNWs. PtNWs were synthesized following a previous procedure[1] with slight modification. Typically, a mixture of KOH (0.6 g) and ethylene glycol (4 mL) was dissolved in DMF (6 mL). Aqueous solution of K_2PtCl_6 (8 wt%, 0.1 mL) was then added into the mixture. After stirring for 20 min, the reaction mixture was transferred into a Teflon-lined autoclave, which was maintained at 150 °C for 15 h and then cooled to room temperature. The black powders were collected after the reaction and washed with ethanol and DI water repeatedly for several times before use.

Preparation of PtNWs films. A free standing film was assembled from as-prepared PtNWs suspension by a co-solvent evaporation method. Typically, PtNWs suspensions in ethanol (400 ul, 0.4 mg/mL) was mixed with DI water (miliQ filtered, 600 uL) and n-Butanol (250 uL). The suspension of PtNWs in mixed solvents was added drop by drop into a flask (about 9 cm in diameter) filled with DI water. A film of PtNWs was then formed on the water surface which was later transferred onto the device.

Fabrication of the PtNWs electrochemical device. A p++ silicon wafer with 300 nm thermal oxide was used as device substrate. Au electrode with Ti adhesion layer (e-beam evaporation) was used for all devices. The fabrication process of PtNWs devices for our electrical spectroscopy measurements are schematically demonstrated in Figure S1. Typically, a poly(methyl methacrylate) (PMMA, A8, MicroChem Corp.) film was prepared by spin-coating on the substrate surface with pre-patterned Au electrodes (Ti/Au, 50nm/50nm). E-beam lithography was then used to open windows on PMMA, which created desired patterns on the substrate. The pre-prepared free standing film of PtNWs was then deposited onto the substrate surface. After the removal of PMMA template, PtNWs was deposited on the device substrate with desired patterns. To eliminate the influence of electrolyte and to avoid electrochemical reactions on the metal electrodes, another layer of PMMA (~500nm thick, electrochemically inert) was then deposited on the PtNWs device with spin-coating. A smaller window that only exposes PtNWs was opened by e-beam lithography. The final device, with exposed PtNWs and PMMA protected electrodes was used for in-device electrochemistry and in situ electrical spectroscopy.

In-device electrochemistry and in situ electrical transport spectroscopy (ETS). The device configuration and measurement set up is schematically illustrated in Supplementary Fig. 1. Ag/AgCl was used as reference electrode (RE) and counter electrode (CE) was prepared by drop-casting ethanol suspension of exfoliated graphene (1mg/mL) onto the glassy carbon electrode (10 μL x5). The capacitance of the stacked graphene on CE is high enough that it behaves as an ideally non-polarizable electrode during the in-device electrochemistry (the faradic current is

small and will introduce negligible change on the potential of the CE). A two channel source-measure unit (SMU, Agilent B2902a) was used for the measurement. The first SMU channel was used as a potentiostat to control the potential of drain electrode as to the reference electrode (V_G), while collecting the current (I_G) through the counter electrode. The connection on SMU as potentiostat is as follows: CE: High Output; RE: High Sense; WE (drain): Low Output short with Low Sense; four-wire configuration was applied during potentiostat measurement. In a typical in-device CV measurement, the scan rate is 32 mV/s. The second SMU channel was used to supply a small potential (50 mV) between Source and Drain electrodes and collecting the corresponding current (I_{SD}). For each device, tens of CV cycles were swept at the beginning of each test to obtain a reproducible and stable device state (Supplementary Fig. 3), before in-device CV and ETS data were acquired and recorded. Moreover, during an in-device CV and ETS measurement, both CV and ETS results were different at the first scan cycle from the following cycles (all following cycle did not differ significantly), therefore, in a typical measurement, two potential cycles were swept and data presented and discussed in this work were obtained from the second scan cycle. For a typical measurement in this study, the Gate/Faradic current is generally several orders of magnitude smaller than the ETS current (I_G ~1nA and I_{SD} ~10μA). Therefore the in-device CV current does not affect the ETS current and no additional background subtraction or other mathematical treatment is needed before the data analysis.

4. Supplementary References

1. Huang, X. *et al.* A Facile Strategy to Pt3Ni Nanocrystals with Highly Porous Features as an Enhanced Oxygen Reduction Reaction Catalyst. *Adv. Mater.* **25**, 2974-2979 (2013).

2. Sondheimer, E. H. The mean free path of electrons in metals. *Adv. Phys.* **50**, 499-537 (2001).

3. Climent, V. & Feliu, J. Thirty years of platinum single crystal electrochemistry. *J Solid State Electrochem* **15**, 1297-1315 (2011).

4. Schiros, T. *et al.* The role of substrate electrons in the wetting of a metal surface. *J. Chem. Phys.* **132**, - (2010).

5. Casalongue, H. S. *et al.* Direct observation of the oxygenated species during oxygen reduction on a platinum fuel cell cathode. *Nat. Commun.* **4**, 2817 (2013).

6. Schiros, T. *et al.* Structure and Bonding of the Water−Hydroxyl Mixed Phase on Pt(111). *J. Phys. Chem. C* **111**, 15003-15012 (2007).

7. Stamenkovic, V. R. *et al.* Improved Oxygen Reduction Activity on Pt3Ni(111) via Increased Surface Site Availability. *Science* **315**, 493-497 (2007).
